# Proposed workflow for the prevention of lead-related superior vena cava syndrome

**DOI:** 10.1016/j.hroo.2026.01.025

**Published:** 2026-01-30

**Authors:** Fenna Daniëls, Erik Lipšic, Hessel F. Groenveld, Michiel Rienstra, Alexander H. Maass

**Affiliations:** 1 Department of Cardiology, University of Groningen, University Medical Center Groningen, Groningen, The Netherlands; 2 Department of Cardiology, Isala Hospital, Zwolle, The Netherlands

**Keywords:** Superior vena cava syndrome, Cardiac implantable electronic device leads, Lead management, Venous access strategy, Venous angiography, Venoplasty

**Key Findings**

▪ Superior vena cava syndrome is an uncommon but clinically significant complication of transvenous cardiac implantable electronic device (CIED) leads and central venous catheters that poses challenges for treatment and future lead management.
▪ We advise avoiding contralateral venous access in patients requiring additional CIED leads or central venous catheters to reduce the risk of superior vena cava syndrome. Instead, venous angiography should be performed to guide the implantation strategy.
▪ Our structured clinical workflow enables tailored venous access strategies, thus minimizing the risk of lead-related superior vena cava syndrome.

## Introduction

In this viewpoint, after an overview of the background of lead-related superior vena cava (SVC) syndrome, we propose a structured clinical workflow intended to reduce the risk of lead-related SVC syndrome. SVC syndrome is a rare condition of stenosis of the SVC resulting in symptoms. In most of 60% of patients, SVC syndrome is caused by a malignancy. However, in approximately 30% of all patients with SVC syndrome, it is related to cardiac implantable electronic device (CIED) leads and central venous catheters (CVCs).^1^ CVCs have their tip at the vena cava–right atrium junction, and common indications include hemodialysis, hemodynamic support, parenteral nutrition, and long-term antibiotic agents. Cardiac leads and CVCs induce endothelial damage, resulting in inflammation and thrombus formation, which can lead to venous stenosis. In case of an insufficient collateral circulation, this can lead to symptoms and thus SVC syndrome.^2^ Symptoms include facial and bilateral upper extremity swelling, distension of vessels in the subcutaneous tissue, headaches, and exercise-induced flushing. However, in patients with stenosis of a lesser degree, the circulation can be sufficient at rest, but insufficient during exercising or in a supine position, thus resulting in symptoms that are only present at specific moments. It is estimated that the prevalence of SVC syndrome in patients with cardiac device leads ranges between 0.6% and 3.5%.^3^^,^^4^ Diagnosis of SVC syndrome can be based on different diagnostic tests, such as venous duplex ultrasonography, computed tomography, or contrast venography. In cases with diagnostic uncertainty, invasive pressure measurements of the SVC and right atrium can also be performed. Especially in patients with stenosis of a lesser degree, measurement of the pressure gradient before and after exercising (physical or pharmacologic) can contribute to the diagnosis. The treatment of lead-associated SVC syndrome differs in clinical practice and is not mentioned in the European Society of Cardiology or American College of Cardiology guidelines. A 2017 Heart Rhythm Society expert consensus on CIED lead management and extraction recommends lead removal.^5^ However, lead extraction is not always feasible and patients usually have an indication for new (transvenous) cardiac device leads or a CVC, which increases the risk of recurrence of SVC syndrome.^6^ Other techniques consist of thrombolysis, mechanical thrombectomy, venoplasty, stent placement, or a combination of these therapies. However, venoplasty can result in recurrent stenosis, leads need to be removed before stenting, thrombolysis is often not successful, and after thrombectomy, there is a risk of recurrent thrombosis.^6^, ^7^, ^8^

In summary, SVC syndrome is a rare but serious complication of transvenous CIED leads and CVC placement, resulting in difficult-to-treat symptoms and posing challenges for future lead management. Therefore, it is better to prevent SVC syndrome than to treat it, which is the focus for the rest of the viewpoint.

## Avoid the contralateral side

Several factors have been associated with an increased risk of venous stenosis and occlusion after CIED implantation, including the presence of multiple cardiac device leads, previous temporary pacing leads, reduced left ventricular ejection fraction, and previous endovascular or pocket infection.^2^ Although unilateral venous occlusion is almost always asymptomatic owing to collateral drainage through the contralateral side, using both the right- and left-sided veins for access may lead to bilateral obstruction and subsequent SVC syndrome. Therefore, we strongly advise avoiding contralateral venous access in patients with a CVC requiring a CIED or patients with a CIED requiring additional electrodes or a CVC.

## Strategy for additional electrodes in patients with CIED

In patients requiring additional CIED electrodes, venous angiography should be performed. In most cases, even with total venous occlusions, additional electrodes can be added after venoplasty.^9^ When venoplasty is not possible, the next step can be lead extraction, where the extracted lead creates a tunnel to enable implantation of a new lead. Moreover, depending on the indication of the CIED, the type of CIED in situ, and the patient’s anatomy, a subcutaneous implantable cardioverter-defibrillator (S-ICD) or leadless pacemaker can also be an option. In case of failure of both venoplasty and lead extraction and in patients in whom S-ICD or leadless pacemakers are not possible, “outside of the box” accesses can be used. For example, reports have shown the feasibility of pacemaker implantation through the femoral and hepatic veins.^10^^,^^11^

## Strategy for nonurgent CVC in patients with CIED

**Figure 1:**
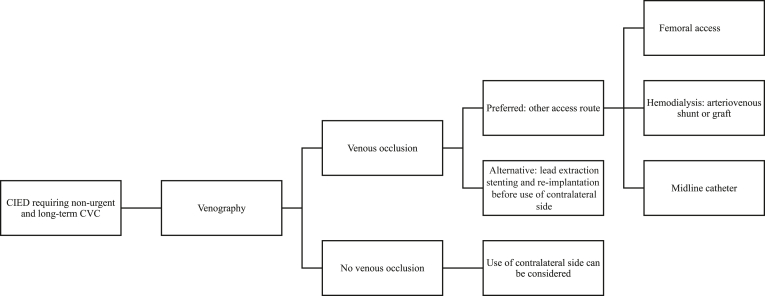
Proposed strategy of CVC implantation in patients with CIED. CIED = cardiac implantable electronic device; CVC = central venous catheter.

In patients with CIEDs requiring a nonurgent and longer-term CVC, we also advise performing venous angiography. Our proposed strategy is shown in Figure 1. When there is no venous occlusion and there is no alternative for the CVC, the contralateral side can be used. In case of venous occlusion with contralateral collaterals, we strongly advise refraining from using the collateral side as venous access and considering other access routes such as the femoral route. For hemodialysis, the possibility of an arteriovenous graft or fistula can be explored. However, grafts or fistulas are not always an option because it takes several weeks to months until these are ready to use for hemodialysis. Alternatively, depending on the indication, a midline catheter can be an option. The midline catheter is usually implanted through the brachial or basilic vein, with the tip in the axillary vein. However, compared with CVCs, midline catheters can be used only for a shorter term of approximately 4 weeks and with less concentrated infusions. Rarely, if all other strategies are contraindicated, lead extraction followed by stenting of the occluded vein and reimplantation of the lead can be the last resolution. However, lead extraction will sacrifice a perfectly working lead, and a risk of recurrent occlusion remains. Moreover, considerable risks are associated with this procedure.

## Strategy in patients with a CVC requiring a new contralateral CVC or CIED lead

**Figure 2:**
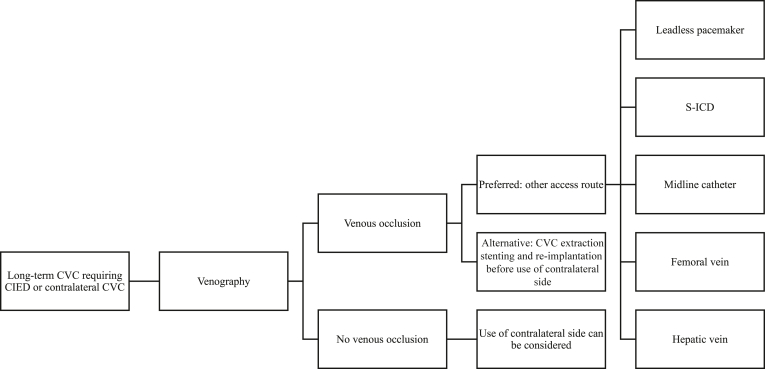
Proposed strategy of CIED implantation or contralateral CVC in patients with long-term CVC. CIED = cardiac implantable electronic device; CVC = central venous catheter; S-ICD = subcutaneous implantable cardioverter-defibrillator.

In patients with a CVC with a definite indication for a new contralateral CVC or a cardiac device lead, again, the first step should be venous angiography. In the absence of venous occlusion, implantation can be pursued. In case of venous occlusion, the previously mentioned other access sites or treatment options (eg, midline, leadless pacemaker, S-ICD, femoral/hepatic veins) can be explored. This is also shown in Figure 2.

## Conclusion

To minimize the risk of SVC syndrome, it is important to perform venous angiography before the implantation of new cardiac device leads or CVCs and adjust the implantation strategy.

## Disclosures

Dr Rienstra reports consultancy fees from Bayer (OCEANIC-AF national principal investigator), InCarda Therapeutics (RESTORE-SR national principal investigator), and Novartis to the institution and speaker fee from Daiichi Sankyo and Pfizer to the institution. Dr Lipšic reports an educational institutional grant from Abbott Medical Nederland BV and a clinical trials grant from Dutch Heart Foundation. The other authors have no conflicts of interest to disclose.
